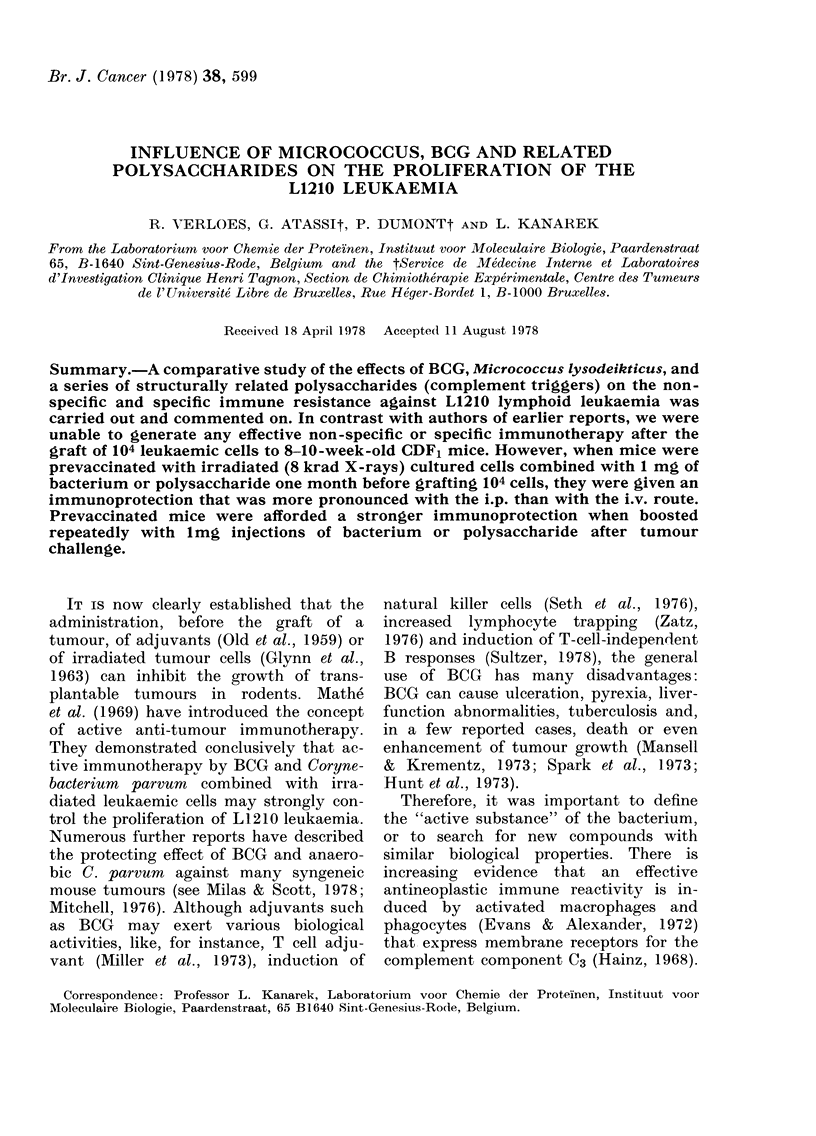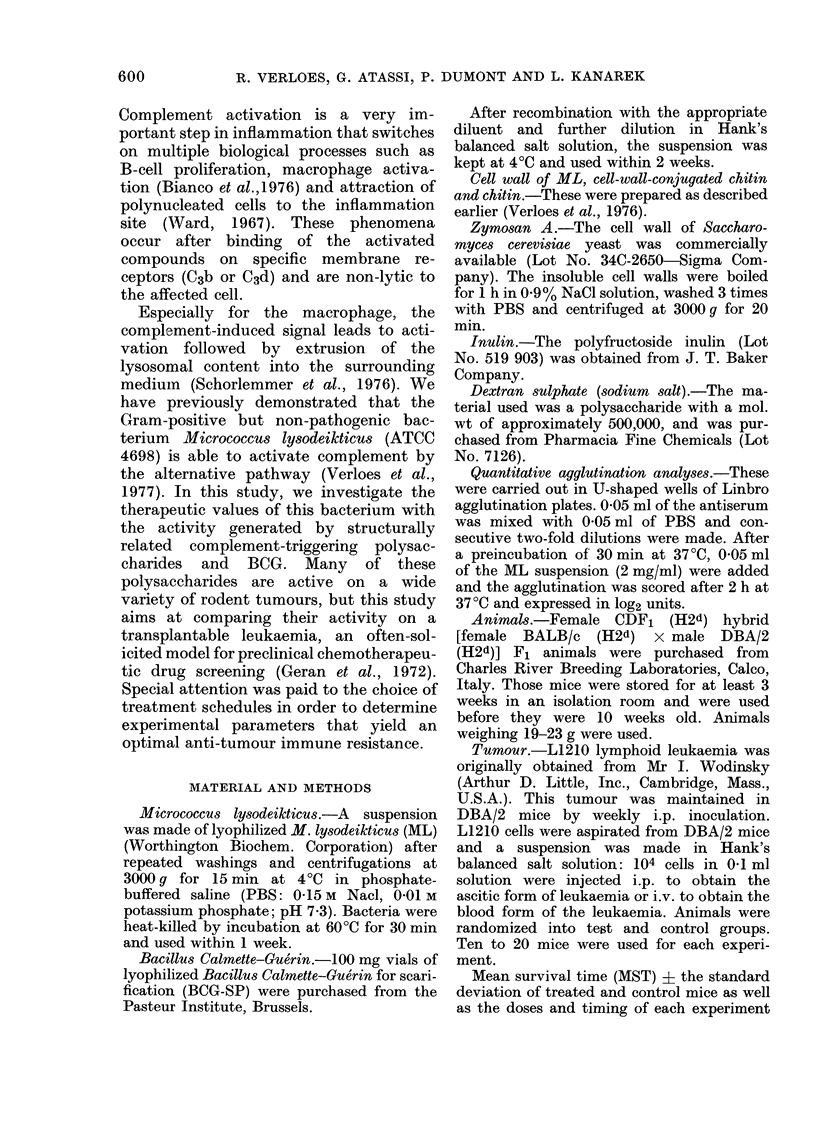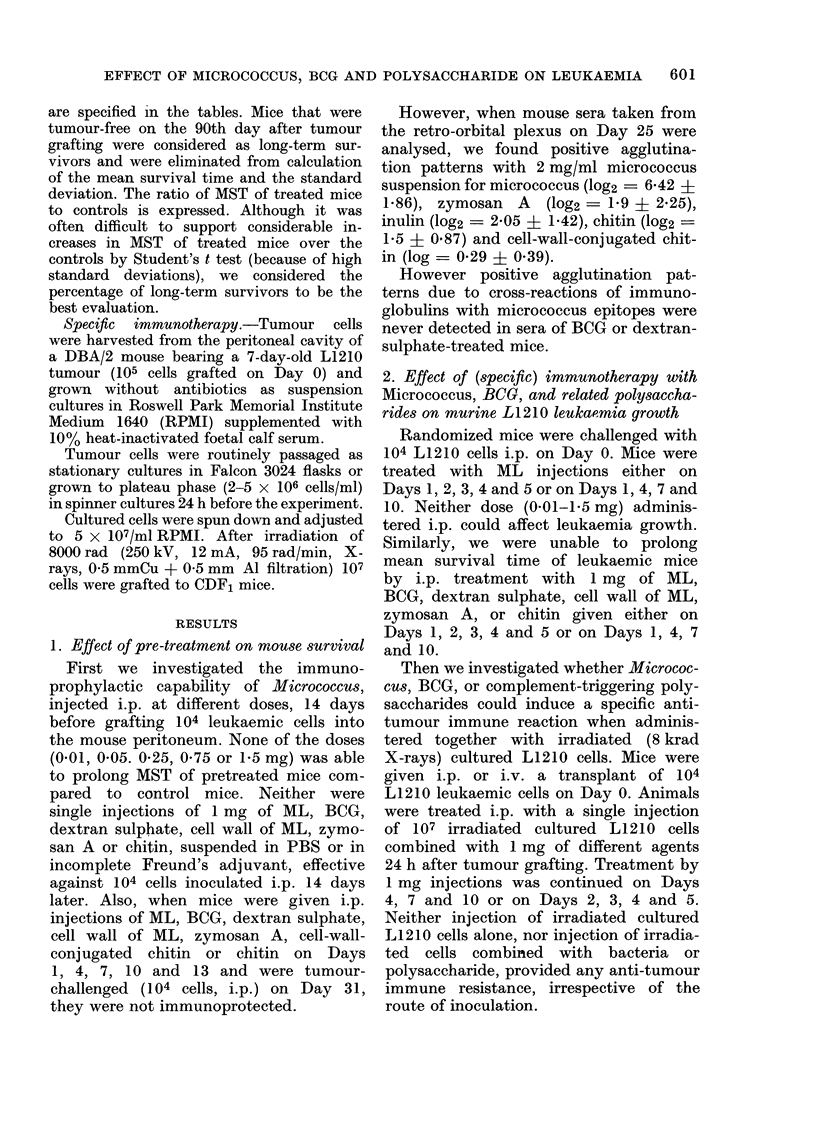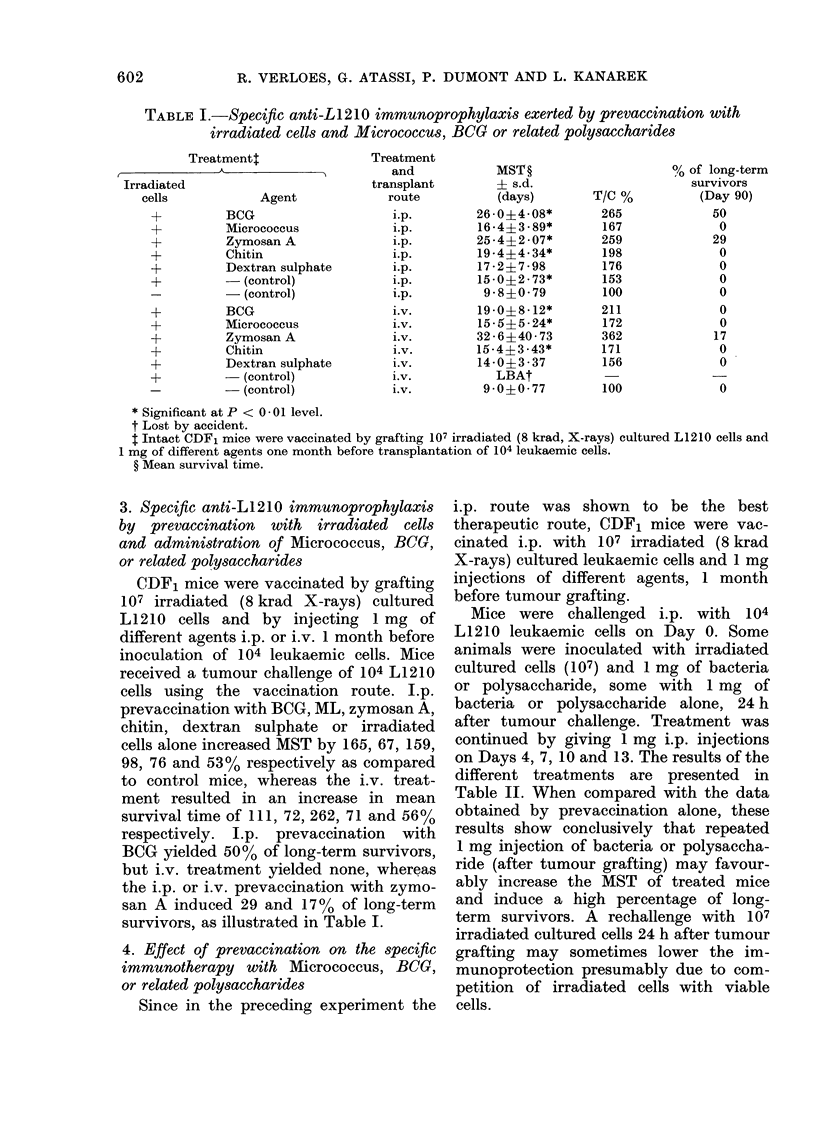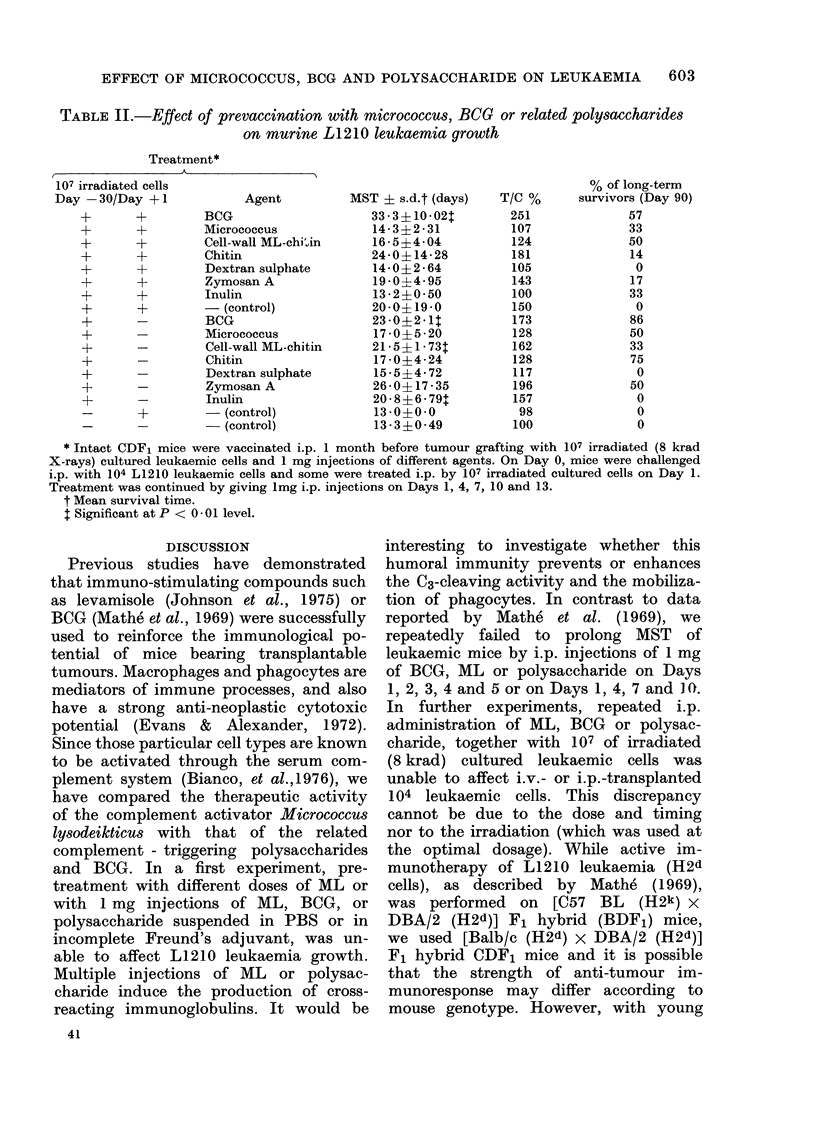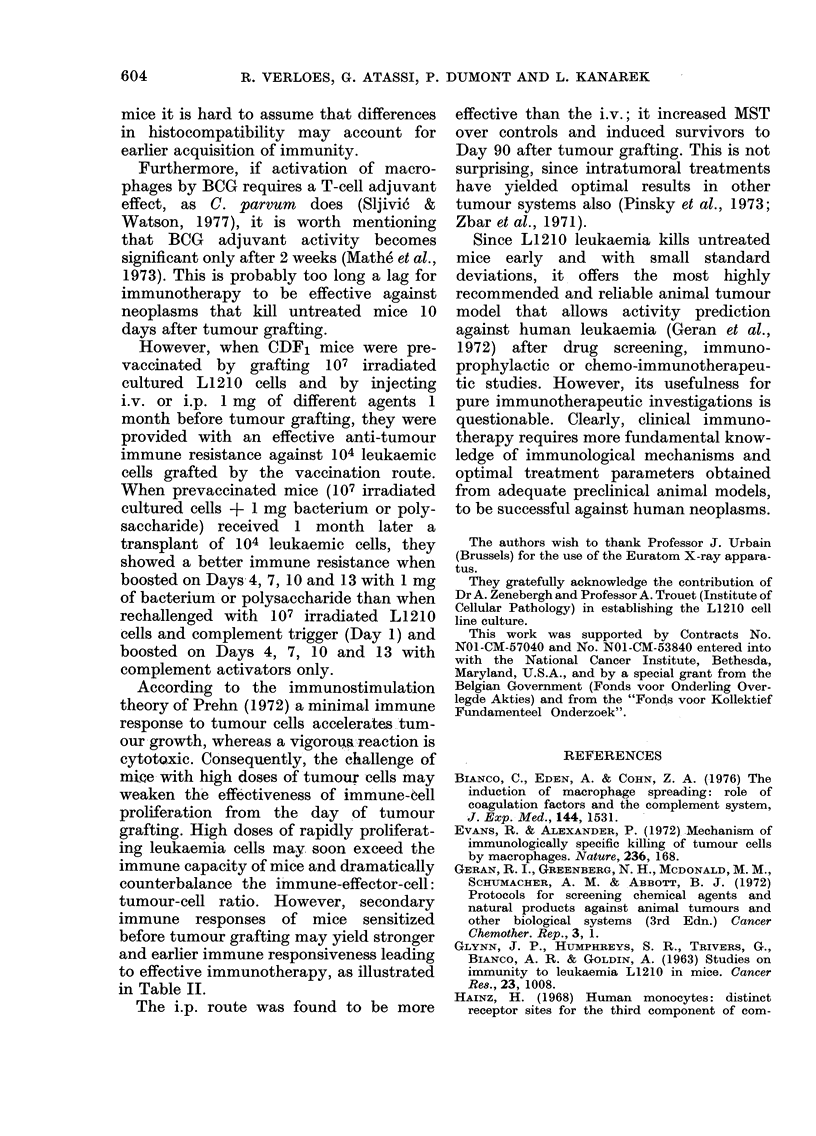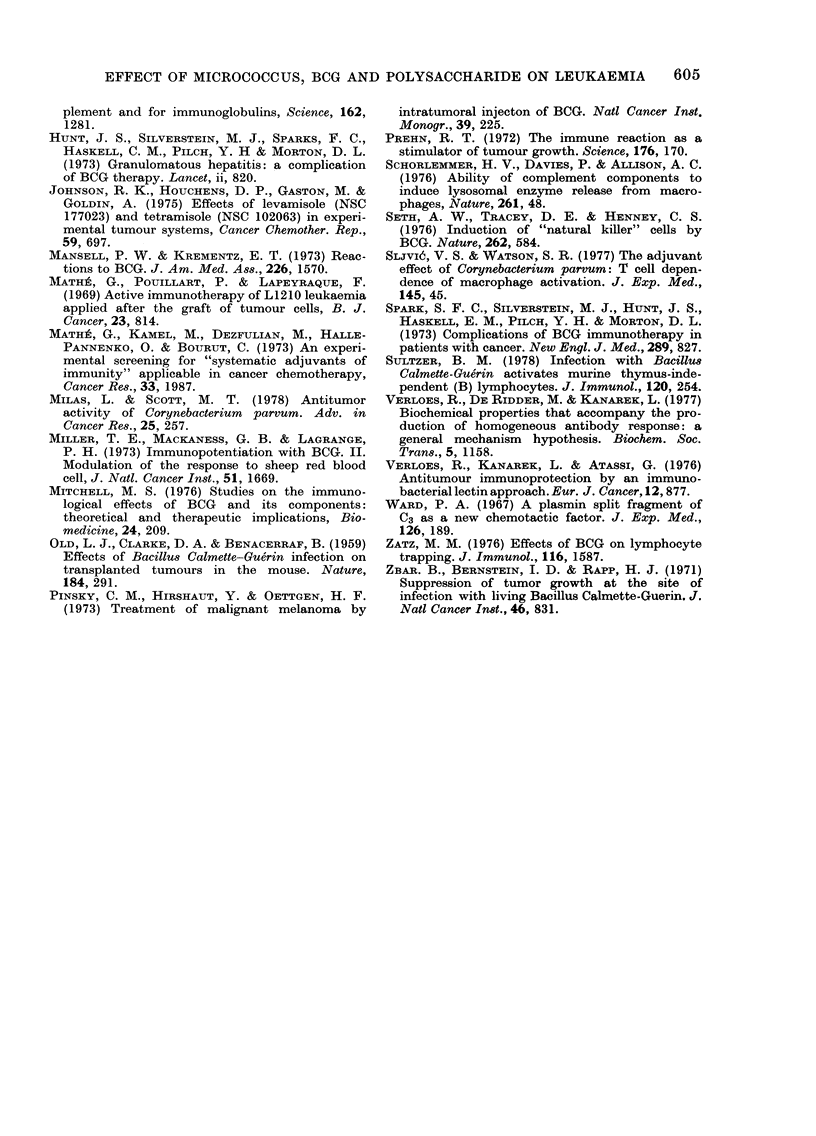# Influence of micrococcus, BCG and related polysaccharides on the proliferation of the L1210 leukaemia.

**DOI:** 10.1038/bjc.1978.255

**Published:** 1978-11

**Authors:** R. Verloes, G. Atassi, P. Dumont, L. Kanarek

## Abstract

A comparative study of the effects of BCG, Micrococcus lysodeikticus, and a series of structurally related polysaccharides (complement triggers) on the non-specific and specific immune resistance against L1210 lymphoid leukaemia was carried out and commented on. In contrast with authors of earlier reports, we were unable to generate any effective non-specific or specific immunotherapy after the graft of 10(4) leukaemic cells to 8--10-week-old CDF1 mice. However, when mice were prevaccinated with irradiated (8 krad X-rays) cultured cells combined with 1 mg of bacterium or polysaccharide one month before grafting 10(4) cells, they were given an immunoprotection that was more pronounced with the i.p. than with the i.v. route. Prevaccinated mice were afforded a stronger immunoprotection when boosted repeatedly with 1mg injections of bacterium or polysaccharide after tumour challenge.


*Br. J. Cancer* (1978) **38**, 599

### INFLUENCE OF MICROCOCCUS, BCG AND RELATED POLYSACCHARIDES ON THE PROLIFERATION OF THE L1210 LEUKAEMIA

R. VERLOES, G. ATASSI†, P. DUMONT† AND L. KANAREK

*From the Laboratorium voor Chemie der Proteïnen, Instituut voor Moleculaire Biologie, Paardenstraat 65, B-1640 Sint-Genesius-Rode, Belgium and the †Service de Médecine Interne et Laboratoires d'Investigation Clinique Henri Tagnon, Section de Chimiothérapie Expérimentale, Centre des Tumeurs de l'Université Libre de Bruxelles, Rue Héger-Bordet 1, B-1000 Bruxelles.*

Received 18 April 1978 Accepted 11 August 1978

**Summary.—A comparative study of the effects of BCG, *Micrococcus lysodeikticus*, and a series of structurally related polysaccharides (complement triggers) on the non-specific and specific immune resistance against L1210 lymphoid leukaemia was carried out and commented on. In contrast with authors of earlier reports, we were unable to generate any effective non-specific or specific immunotherapy after the graft of $10^4$ leukaemic cells to 8–10-week-old $CDF_1$ mice. However, when mice were prevaccinated with irradiated (8 krad X-rays) cultured cells combined with 1 mg of bacterium or polysaccharide one month before grafting $10^4$ cells, they were given an immunoprotection that was more pronounced with the i.p. than with the i.v. route. Prevaccinated mice were afforded a stronger immunoprotection when boosted repeatedly with 1mg injections of bacterium or polysaccharide after tumour challenge.**

It is now clearly established that the administration, before the graft of a tumour, of adjuvants (Old *et al.*, 1959) or of irradiated tumour cells (Glynn *et al.*, 1963) can inhibit the growth of transplantable tumours in rodents. Mathé *et al.* (1969) have introduced the concept of active anti-tumour immunotherapy. They demonstrated conclusively that active immunotherapy by BCG and *Corynebacterium parvum* combined with irradiated leukaemic cells may strongly control the proliferation of L1210 leukaemia. Numerous further reports have described the protecting effect of BCG and anaerobic *C. parvum* against many syngeneic mouse tumours (see Milas & Scott, 1978; Mitchell, 1976). Although adjuvants such as BCG may exert various biological activities, like, for instance, T cell adjuvant (Miller *et al.*, 1973), induction of natural killer cells (Seth *et al.*, 1976), increased lymphocyte trapping (Zatz, 1976) and induction of T-cell-independent B responses (Sultzer, 1978), the general use of BCG has many disadvantages: BCG can cause ulceration, pyrexia, liver-function abnormalities, tuberculosis and, in a few reported cases, death or even enhancement of tumour growth (Mansell & Krementz, 1973; Spark *et al.*, 1973; Hunt *et al.*, 1973).

Therefore, it was important to define the "active substance" of the bacterium, or to search for new compounds with similar biological properties. There is increasing evidence that an effective antineoplastic immune reactivity is induced by activated macrophages and phagocytes (Evans & Alexander, 1972) that express membrane receptors for the complement component $C_3$ (Hainz, 1968).

Correspondence: Professor L. Kanarek, Laboratorium voor Chemie der Proteïnen, Instituut voor Moleculaire Biologie, Paardenstraat, 65 B1640 Sint-Genesius-Rode, Belgium.

Complement activation is a very important step in inflammation that switches on multiple biological processes such as B-cell proliferation, macrophage activation (Bianco *et al.*,1976) and attraction of polynucleated cells to the inflammation site (Ward, 1967). These phenomena occur after binding of the activated compounds on specific membrane receptors ($C_3b$ or $C_3d$) and are non-lytic to the affected cell.

Especially for the macrophage, the complement-induced signal leads to activation followed by extrusion of the lysosomal content into the surrounding medium (Schorlemmer *et al.*, 1976). We have previously demonstrated that the Gram-positive but non-pathogenic bacterium *Micrococcus lysodeikticus* (ATCC 4698) is able to activate complement by the alternative pathway (Verloes *et al.*, 1977). In this study, we investigate the therapeutic values of this bacterium with the activity generated by structurally related complement-triggering polysaccharides and BCG. Many of these polysaccharides are active on a wide variety of rodent tumours, but this study aims at comparing their activity on a transplantable leukaemia, an often-solicited model for preclinical chemotherapeutic drug screening (Geran *et al.*, 1972). Special attention was paid to the choice of treatment schedules in order to determine experimental parameters that yield an optimal anti-tumour immune resistance.

### MATERIAL AND METHODS

*Micrococcus lysodeikticus*.—A suspension was made of lyophilized *M. lysodeikticus* (ML) (Worthington Biochem. Corporation) after repeated washings and centrifugations at 3000 *g* for 15 min at 4°C in phosphate-buffered saline (PBS: 0·15 M Nacl, 0·01 M potassium phosphate; pH 7·3). Bacteria were heat-killed by incubation at 60°C for 30 min and used within 1 week.

*Bacillus Calmette–Guérin*.—100 mg vials of lyophilized *Bacillus Calmette–Guérin* for scarification (BCG-SP) were purchased from the Pasteur Institute, Brussels.

After recombination with the appropriate diluent and further dilution in Hank's balanced salt solution, the suspension was kept at 4°C and used within 2 weeks.

*Cell wall of ML, cell-wall-conjugated chitin and chitin*.—These were prepared as described earlier (Verloes *et al.*, 1976).

*Zymosan A*.—The cell wall of *Saccharomyces cerevisiae* yeast was commercially available (Lot No. 34C-2650—Sigma Company). The insoluble cell walls were boiled for 1 h in 0·9% NaCl solution, washed 3 times with PBS and centrifuged at 3000 *g* for 20 min.

*Inulin*.—The polyfructoside inulin (Lot No. 519 903) was obtained from J. T. Baker Company.

*Dextran sulphate* (*sodium salt*).—The material used was a polysaccharide with a mol. wt of approximately 500,000, and was purchased from Pharmacia Fine Chemicals (Lot No. 7126).

*Quantitative agglutination analyses*.—These were carried out in U-shaped wells of Linbro agglutination plates. 0·05 ml of the antiserum was mixed with 0·05 ml of PBS and consecutive two-fold dilutions were made. After a preincubation of 30 min at 37°C, 0·05 ml of the ML suspension (2 mg/ml) were added and the agglutination was scored after 2 h at 37°C and expressed in $\log_2$ units.

*Animals*.—Female $CDF_1$ ($H2^d$) hybrid [female BALB/c ($H2^d$) × male DBA/2 ($H2^d$)] $F_1$ animals were purchased from Charles River Breeding Laboratories, Calco, Italy. Those mice were stored for at least 3 weeks in an isolation room and were used before they were 10 weeks old. Animals weighing 19–23 g were used.

*Tumour*.—L1210 lymphoid leukaemia was originally obtained from Mr I. Wodinsky (Arthur D. Little, Inc., Cambridge, Mass., U.S.A.). This tumour was maintained in DBA/2 mice by weekly i.p. inoculation. L1210 cells were aspirated from DBA/2 mice and a suspension was made in Hank's balanced salt solution: $10^4$ cells in 0·1 ml solution were injected i.p. to obtain the ascitic form of leukaemia or i.v. to obtain the blood form of the leukaemia. Animals were randomized into test and control groups. Ten to 20 mice were used for each experiment.

Mean survival time (MST) ± the standard deviation of treated and control mice as well as the doses and timing of each experiment are specified in the tables. Mice that were tumour-free on the 90th day after tumour grafting were considered as long-term survivors and were eliminated from calculation of the mean survival time and the standard deviation. The ratio of MST of treated mice to controls is expressed. Although it was often difficult to support considerable increases in MST of treated mice over the controls by Student's $t$ test (because of high standard deviations), we considered the percentage of long-term survivors to be the best evaluation.

*Specific immunotherapy.*—Tumour cells were harvested from the peritoneal cavity of a DBA/2 mouse bearing a 7-day-old L1210 tumour ($10^5$ cells grafted on Day 0) and grown without antibiotics as suspension cultures in Roswell Park Memorial Institute Medium 1640 (RPMI) supplemented with 10% heat-inactivated foetal calf serum.

Tumour cells were routinely passaged as stationary cultures in Falcon 3024 flasks or grown to plateau phase (2–5 $\times$ $10^6$ cells/ml) in spinner cultures 24 h before the experiment.

Cultured cells were spun down and adjusted to 5 $\times$ $10^7$/ml RPMI. After irradiation of 8000 rad (250 kV, 12 mA, 95 rad/min, X-rays, 0·5 mmCu + 0·5 mm Al filtration) $10^7$ cells were grafted to $CDF_1$ mice.

### RESULTS

### 1. *Effect of pre-treatment on mouse survival*

First we investigated the immunoprophylactic capability of *Micrococcus*, injected i.p. at different doses, 14 days before grafting $10^4$ leukaemic cells into the mouse peritoneum. None of the doses (0·01, 0·05. 0·25, 0·75 or 1·5 mg) was able to prolong MST of pretreated mice compared to control mice. Neither were single injections of 1 mg of ML, BCG, dextran sulphate, cell wall of ML, zymosan A or chitin, suspended in PBS or in incomplete Freund's adjuvant, effective against $10^4$ cells inoculated i.p. 14 days later. Also, when mice were given i.p. injections of ML, BCG, dextran sulphate, cell wall of ML, zymosan A, cell-wall-conjugated chitin or chitin on Days 1, 4, 7, 10 and 13 and were tumour-challenged ($10^4$ cells, i.p.) on Day 31, they were not immunoprotected.

However, when mouse sera taken from the retro-orbital plexus on Day 25 were analysed, we found positive agglutination patterns with 2 mg/ml micrococcus suspension for micrococcus ($\log_2 = 6{\cdot}42 \pm 1{\cdot}86$), zymosan A ($\log_2 = 1{\cdot}9 \pm 2{\cdot}25$), inulin ($\log_2 = 2{\cdot}05 \pm 1{\cdot}42$), chitin ($\log_2 = 1{\cdot}5 \pm 0{\cdot}87$) and cell-wall-conjugated chitin ($\log = 0{\cdot}29 \pm 0{\cdot}39$).

However positive agglutination patterns due to cross-reactions of immunoglobulins with micrococcus epitopes were never detected in sera of BCG or dextran-sulphate-treated mice.

### 2. *Effect of (specific) immunotherapy with* Micrococcus, *BCG, and related polysaccharides on murine L1210 leukaemia growth*

Randomized mice were challenged with $10^4$ L1210 cells i.p. on Day 0. Mice were treated with ML injections either on Days 1, 2, 3, 4 and 5 or on Days 1, 4, 7 and 10. Neither dose (0·01–1·5 mg) administered i.p. could affect leukaemia growth. Similarly, we were unable to prolong mean survival time of leukaemic mice by i.p. treatment with 1 mg of ML, BCG, dextran sulphate, cell wall of ML, zymosan A, or chitin given either on Days 1, 2, 3, 4 and 5 or on Days 1, 4, 7 and 10.

Then we investigated whether *Micrococcus*, BCG, or complement-triggering polysaccharides could induce a specific anti-tumour immune reaction when administered together with irradiated (8 krad X-rays) cultured L1210 cells. Mice were given i.p. or i.v. a transplant of $10^4$ L1210 leukaemic cells on Day 0. Animals were treated i.p. with a single injection of $10^7$ irradiated cultured L1210 cells combined with 1 mg of different agents 24 h after tumour grafting. Treatment by 1 mg injections was continued on Days 4, 7 and 10 or on Days 2, 3, 4 and 5. Neither injection of irradiated cultured L1210 cells alone, nor injection of irradiated cells combined with bacteria or polysaccharide, provided any anti-tumour immune resistance, irrespective of the route of inoculation.

TABLE I.—*Specific anti-L1210 immunoprophylaxis exerted by prevaccination with irradiated cells and Micrococcus, BCG or related polysaccharides*

| Treatment‡ Irradiated cells | Treatment‡ Agent | Treatment and transplant route | MST§ ± s.d. (days) | T/C % | % of long-term survivors (Day 90) |
|---|---|---|---|---|---|
| + | BCG | i.p. | 26·0±4·08* | 265 | 50 |
| + | Micrococcus | i.p. | 16·4±3·89* | 167 | 0 |
| + | Zymosan A | i.p. | 25·4±2·07* | 259 | 29 |
| + | Chitin | i.p. | 19·4±4·34* | 198 | 0 |
| + | Dextran sulphate | i.p. | 17·2±7·98 | 176 | 0 |
| + | — (control) | i.p. | 15·0±2·73* | 153 | 0 |
| — | — (control) | i.p. | 9·8±0·79 | 100 | 0 |
| + | BCG | i.v. | 19·0±8·12* | 211 | 0 |
| + | Micrococcus | i.v. | 15·5±5·24* | 172 | 0 |
| + | Zymosan A | i.v. | 32·6±40·73 | 362 | 17 |
| + | Chitin | i.v. | 15·4±3·43* | 171 | 0 |
| + | Dextran sulphate | i.v. | 14·0±3·37 | 156 | 0 |
| + | — (control) | i.v. | LBA† | — | — |
| — | — (control) | i.v. | 9·0±0·77 | 100 | 0 |

* Significant at $P < 0{\cdot}01$ level.
† Lost by accident.
‡ Intact $CDF_1$ mice were vaccinated by grafting $10^7$ irradiated (8 krad, X-rays) cultured L1210 cells and 1 mg of different agents one month before transplantation of $10^4$ leukaemic cells.
§ Mean survival time.

3. *Specific anti-*L1210 *immunoprophylaxis by prevaccination with irradiated cells and administration of* Micrococcus, *BCG, or related polysaccharides*

$CDF_1$ mice were vaccinated by grafting $10^7$ irradiated (8 krad X-rays) cultured L1210 cells and by injecting 1 mg of different agents i.p. or i.v. 1 month before inoculation of $10^4$ leukaemic cells. Mice received a tumour challenge of $10^4$ L1210 cells using the vaccination route. I.p. prevaccination with BCG, ML, zymosan A, chitin, dextran sulphate or irradiated cells alone increased MST by 165, 67, 159, 98, 76 and 53% respectively as compared to control mice, whereas the i.v. treatment resulted in an increase in mean survival time of 111, 72, 262, 71 and 56% respectively. I.p. prevaccination with BCG yielded 50% of long-term survivors, but i.v. treatment yielded none, whereas the i.p. or i.v. prevaccination with zymosan A induced 29 and 17% of long-term survivors, as illustrated in Table I.

4. *Effect of prevaccination on the specific immunotherapy with* Micrococcus, *BCG, or related polysaccharides*

Since in the preceding experiment the i.p. route was shown to be the best therapeutic route, $CDF_1$ mice were vaccinated i.p. with $10^7$ irradiated (8 krad X-rays) cultured leukaemic cells and 1 mg injections of different agents, 1 month before tumour grafting.

Mice were challenged i.p. with $10^4$ L1210 leukaemic cells on Day 0. Some animals were inoculated with irradiated cultured cells ($10^7$) and 1 mg of bacteria or polysaccharide, some with 1 mg of bacteria or polysaccharide alone, 24 h after tumour challenge. Treatment was continued by giving 1 mg i.p. injections on Days 4, 7, 10 and 13. The results of the different treatments are presented in Table II. When compared with the data obtained by prevaccination alone, these results show conclusively that repeated 1 mg injection of bacteria or polysaccharide (after tumour grafting) may favourably increase the MST of treated mice and induce a high percentage of long-term survivors. A rechallenge with $10^7$ irradiated cultured cells 24 h after tumour grafting may sometimes lower the immunoprotection presumably due to competition of irradiated cells with viable cells.

TABLE II.—*Effect of prevaccination with micrococcus, BCG or related polysaccharides on murine L1210 leukaemia growth*

| Treatment* $10^7$ irradiated cells Day −30 | Day +1 | Agent | MST ± s.d.† (days) | T/C % | % of long-term survivors (Day 90) |
|---|---|---|---|---|---|
| + | + | BCG | 33·3±10·02‡ | 251 | 57 |
| + | + | Micrococcus | 14·3±2·31 | 107 | 33 |
| + | + | Cell-wall ML-chitin | 16·5±4·04 | 124 | 50 |
| + | + | Chitin | 24·0±14·28 | 181 | 14 |
| + | + | Dextran sulphate | 14·0±2·64 | 105 | 0 |
| + | + | Zymosan A | 19·0±4·95 | 143 | 17 |
| + | + | Inulin | 13·2±0·50 | 100 | 33 |
| + | + | — (control) | 20·0±19·0 | 150 | 0 |
| + | − | BCG | 23·0±2·1‡ | 173 | 86 |
| + | − | Micrococcus | 17·0±5·20 | 128 | 50 |
| + | − | Cell-wall ML-chitin | 21·5±1·73‡ | 162 | 33 |
| + | − | Chitin | 17·0±4·24 | 128 | 75 |
| + | − | Dextran sulphate | 15·5±4·72 | 117 | 0 |
| + | − | Zymosan A | 26·0±17·35 | 196 | 50 |
| + | − | Inulin | 20·8±6·79‡ | 157 | 0 |
| − | + | — (control) | 13·0±0·0 | 98 | 0 |
| − | − | — (control) | 13·3±0·49 | 100 | 0 |

* Intact $CDF_1$ mice were vaccinated i.p. 1 month before tumour grafting with $10^7$ irradiated (8 krad X-rays) cultured leukaemic cells and 1 mg injections of different agents. On Day 0, mice were challenged i.p. with $10^4$ L1210 leukaemic cells and some were treated i.p. by $10^7$ irradiated cultured cells on Day 1. Treatment was continued by giving 1mg i.p. injections on Days 1, 4, 7, 10 and 13.

† Mean survival time.

‡ Significant at $P < 0 \cdot 01$ level.

### DISCUSSION

Previous studies have demonstrated that immuno-stimulating compounds such as levamisole (Johnson *et al.*, 1975) or BCG (Mathé *et al.*, 1969) were successfully used to reinforce the immunological potential of mice bearing transplantable tumours. Macrophages and phagocytes are mediators of immune processes, and also have a strong anti-neoplastic cytotoxic potential (Evans & Alexander, 1972). Since those particular cell types are known to be activated through the serum complement system (Bianco, *et al.*,1976), we have compared the therapeutic activity of the complement activator *Micrococcus lysodeikticus* with that of the related complement - triggering polysaccharides and BCG. In a first experiment, pretreatment with different doses of ML or with 1 mg injections of ML, BCG, or polysaccharide suspended in PBS or in incomplete Freund's adjuvant, was unable to affect L1210 leukaemia growth. Multiple injections of ML or polysaccharide induce the production of cross-reacting immunoglobulins. It would be interesting to investigate whether this humoral immunity prevents or enhances the $C_3$-cleaving activity and the mobilization of phagocytes. In contrast to data reported by Mathé *et al.* (1969), we repeatedly failed to prolong MST of leukaemic mice by i.p. injections of 1 mg of BCG, ML or polysaccharide on Days 1, 2, 3, 4 and 5 or on Days 1, 4, 7 and 10. In further experiments, repeated i.p. administration of ML, BCG or polysaccharide, together with $10^7$ of irradiated (8 krad) cultured leukaemic cells was unable to affect i.v.- or i.p.-transplanted $10^4$ leukaemic cells. This discrepancy cannot be due to the dose and timing nor to the irradiation (which was used at the optimal dosage). While active immunotherapy of L1210 leukaemia ($H2^d$ cells), as described by Mathé (1969), was performed on [C57 BL ($H2^k$) × DBA/2 ($H2^d$)] $F_1$ hybrid ($BDF_1$) mice, we used [Balb/c ($H2^d$) × DBA/2 ($H2^d$)] $F_1$ hybrid $CDF_1$ mice and it is possible that the strength of anti-tumour immunoresponse may differ according to mouse genotype. However, with young mice it is hard to assume that differences in histocompatibility may account for earlier acquisition of immunity.

Furthermore, if activation of macrophages by BCG requires a T-cell adjuvant effect, as *C. parvum* does (Sljivić & Watson, 1977), it is worth mentioning that BCG adjuvant activity becomes significant only after 2 weeks (Mathé *et al.*, 1973). This is probably too long a lag for immunotherapy to be effective against neoplasms that kill untreated mice 10 days after tumour grafting.

However, when $CDF_1$ mice were prevaccinated by grafting $10^7$ irradiated cultured L1210 cells and by injecting i.v. or i.p. 1 mg of different agents 1 month before tumour grafting, they were provided with an effective anti-tumour immune resistance against $10^4$ leukaemic cells grafted by the vaccination route. When prevaccinated mice ($10^7$ irradiated cultured cells + 1 mg bacterium or polysaccharide) received 1 month later a transplant of $10^4$ leukaemic cells, they showed a better immune resistance when boosted on Days 4, 7, 10 and 13 with 1 mg of bacterium or polysaccharide than when rechallenged with $10^7$ irradiated L1210 cells and complement trigger (Day 1) and boosted on Days 4, 7, 10 and 13 with complement activators only.

According to the immunostimulation theory of Prehn (1972) a minimal immune response to tumour cells accelerates tumour growth, whereas a vigorous reaction is cytotoxic. Consequently, the challenge of mice with high doses of tumour cells may weaken the effectiveness of immune-cell proliferation from the day of tumour grafting. High doses of rapidly proliferating leukaemia cells may soon exceed the immune capacity of mice and dramatically counterbalance the immune-effector-cell: tumour-cell ratio. However, secondary immune responses of mice sensitized before tumour grafting may yield stronger and earlier immune responsiveness leading to effective immunotherapy, as illustrated in Table II.

The i.p. route was found to be more effective than the i.v.; it increased MST over controls and induced survivors to Day 90 after tumour grafting. This is not surprising, since intratumoral treatments have yielded optimal results in other tumour systems also (Pinsky *et al.*, 1973; Zbar *et al.*, 1971).

Since L1210 leukaemia kills untreated mice early and with small standard deviations, it offers the most highly recommended and reliable animal tumour model that allows activity prediction against human leukaemia (Geran *et al.*, 1972) after drug screening, immunoprophylactic or chemo-immunotherapeutic studies. However, its usefulness for pure immunotherapeutic investigations is questionable. Clearly, clinical immunotherapy requires more fundamental knowledge of immunological mechanisms and optimal treatment parameters obtained from adequate preclinical animal models, to be successful against human neoplasms.

The authors wish to thank Professor J. Urbain (Brussels) for the use of the Euratom X-ray apparatus.

They gratefully acknowledge the contribution of Dr A. Zenebergh and Professor A. Trouet (Institute of Cellular Pathology) in establishing the L1210 cell line culture.

This work was supported by Contracts No. N01-CM-57040 and No. N01-CM-53840 entered into with the National Cancer Institute, Bethesda, Maryland, U.S.A., and by a special grant from the Belgian Government (Fonds voor Onderling Overlegde Akties) and from the "Fonds voor Kollektief Fundamenteel Onderzoek".

### REFERENCES

Bianco, C., Eden, A. & Cohn, Z. A. (1976) The induction of macrophage spreading: role of coagulation factors and the complement system, *J. Exp. Med.*, **144**, 1531.

Evans, R. & Alexander, P. (1972) Mechanism of immunologically specific killing of tumour cells by macrophages. *Nature*, **236**, 168.

Geran, R. I., Greenberg, N. H., Mcdonald, M. M., Schumacher, A. M. & Abbott, B. J. (1972) Protocols for screening chemical agents and natural products against animal tumours and other biological systems (3rd Edn.) *Cancer Chemother. Rep.*, **3**, 1.

Glynn, J. P., Humphreys, S. R., Trivers, G., Bianco, A. R. & Goldin, A. (1963) Studies on immunity to leukaemia L1210 in mice. *Cancer Res.*, **23**, 1008.

Hainz, H. (1968) Human monocytes: distinct receptor sites for the third component of complement and for immunoglobulins, *Science*, **162**, 1281.

HUNT, J. S., SILVERSTEIN, M. J., SPARKS, F. C., HASKELL, C. M., PILCH, Y. H & MORTON, D. L. (1973) Granulomatous hepatitis: a complication of BCG therapy. *Lancet*, ii, 820.

JOHNSON, R. K., HOUCHENS, D. P., GASTON, M. & GOLDIN, A. (1975) Effects of levamisole (NSC 177023) and tetramisole (NSC 102063) in experimental tumour systems, *Cancer Chemother. Rep.*, **59**, 697.

MANSELL, P. W. & KREMENTZ, E. T. (1973) Reactions to BCG. *J. Am. Med. Ass.*, **226**, 1570.

MATHÉ, G., POUILLART, P. & LAPEYRAQUE, F. (1969) Active immunotherapy of L1210 leukaemia applied after the graft of tumour cells, *B. J. Cancer*, **23**, 814.

MATHÉ, G., KAMEL, M., DEZFULIAN, M., HALLE-PANNENKO, O. & BOURUT, C. (1973) An experimental screening for "systematic adjuvants of immunity" applicable in cancer chemotherapy, *Cancer Res.*, **33**, 1987.

MILAS, L. & SCOTT, M. T. (1978) Antitumor activity of *Corynebacterium parvum*. *Adv. in Cancer Res.*, **25**, 257.

MILLER, T. E., MACKANESS, G. B. & LAGRANGE, P. H. (1973) Immunopotentiation with BCG. II. Modulation of the response to sheep red blood cell, *J. Natl. Cancer Inst.*, **51**, 1669.

MITCHELL, M. S. (1976) Studies on the immunological effects of BCG and its components: theoretical and therapeutic implications, *Biomedicine*, **24**, 209.

OLD, L. J., CLARKE, D. A. & BENACERRAF, B. (1959) Effects of *Bacillus Calmette–Guérin* infection on transplanted tumours in the mouse. *Nature*, **184**, 291.

PINSKY, C. M., HIRSHAUT, Y. & OETTGEN, H. F. (1973) Treatment of malignant melanoma by intratumoral injecton of BCG. *Natl Cancer Inst. Monogr.*, **39**, 225.

PREHN, R. T. (1972) The immune reaction as a stimulator of tumour growth. *Science*, **176**, 170.

SCHORLEMMER, H. V., DAVIES, P. & ALLISON, A. C. (1976) Ability of complement components to induce lysosomal enzyme release from macrophages, *Nature*, **261**, 48.

SETH, A. W., TRACEY, D. E. & HENNEY, C. S. (1976) Induction of "natural killer" cells by BCG. *Nature*, **262**, 584.

SLJVIĆ, V. S. & WATSON, S. R. (1977) The adjuvant effect of *Corynebacterium parvum*: T cell dependence of macrophage activation. *J. Exp. Med.*, **145**, 45.

SPARK, S. F. C., SILVERSTEIN, M. J., HUNT, J. S., HASKELL, E. M., PILCH, Y. H. & MORTON, D. L. (1973) Complications of BCG immunotherapy in patients with cancer. *New Engl. J. Med.*, **289**, 827.

SULTZER, B. M. (1978) Infection with *Bacillus Calmette-Guérin* activates murine thymus-independent (B) lymphocytes. *J. Immunol.*, **120**, 254.

VERLOES, R., DE RIDDER, M. & KANAREK, L. (1977) Biochemical properties that accompany the production of homogeneous antibody response: a general mechanism hypothesis. *Biochem. Soc. Trans.*, **5**, 1158.

VERLOES, R., KANAREK, L. & ATASSI, G. (1976) Antitumour immunoprotection by an immunobacterial lectin approach. *Eur. J. Cancer*, **12**, 877.

WARD, P. A. (1967) A plasmin split fragment of $C_3$ as a new chemotactic factor. *J. Exp. Med.*, **126**, 189.

ZATZ, M. M. (1976) Effects of BCG on lymphocyte trapping. *J. Immunol.*, **116**, 1587.

ZBAR. B., BERNSTEIN, I. D. & RAPP, H. J. (1971) Suppression of tumor growth at the site of infection with living Bacillus Calmette-Guerin. *J. Natl Cancer Inst.*, **46**, 831.